# Supplementary material for: Phosphatidylserine receptors enhance SARS-CoV-2 infection
Source: PLoS Pathog. 2021 Nov 19;17(11):e1009743. doi: 10.1371/journal.ppat.1009743 (PMC8641883; doi:10.1371/journal.ppat.1009743)
Supplement: S1 Fig — A) Representative surface staining of receptors transfected into cells. B) Surface expression (MFI) of proteins in mock transfected (empty vector) and transfected HEK 293T at 48 hours after transfection. Background fluorescence is shown for secondary antibodies used in experiment (α-goat or rabbit secondaries). C) HEK 293T cells, transfected PS receptors as noted with or without 250 ng of ACE2 were transduced with rVSV/Spike. Transduction was assessed 24 hours later via luminescence. D) Expression of MerTK did not affect rVSV/Spike transduction in the presence of 250 ng of transfected ACE2 plasmid. E) Expression of ACE2, TIM-1 or AXL did not enhance infection of VSV-luciferase/Lassa virus GP pseudovirions. HEK 293T cells were transfected with PS receptor plasmids and 50 ng of ACE2 and infected 48 hours later. Panels C, D, and E are shown as fold change of luciferase activity in cell lysates relative to mock transfected lysates that were set to a value of 1. Data shown are pooled from at least three independent experiments (C, D and E). Data represented as means ± SEM. One-Way ANOVA with multiple comparisons (C, E), Student’s t-test (D); asterisks represent p < 0.05. (PDF) [file ppat.1009743.s001.pdf]

S1 Fig

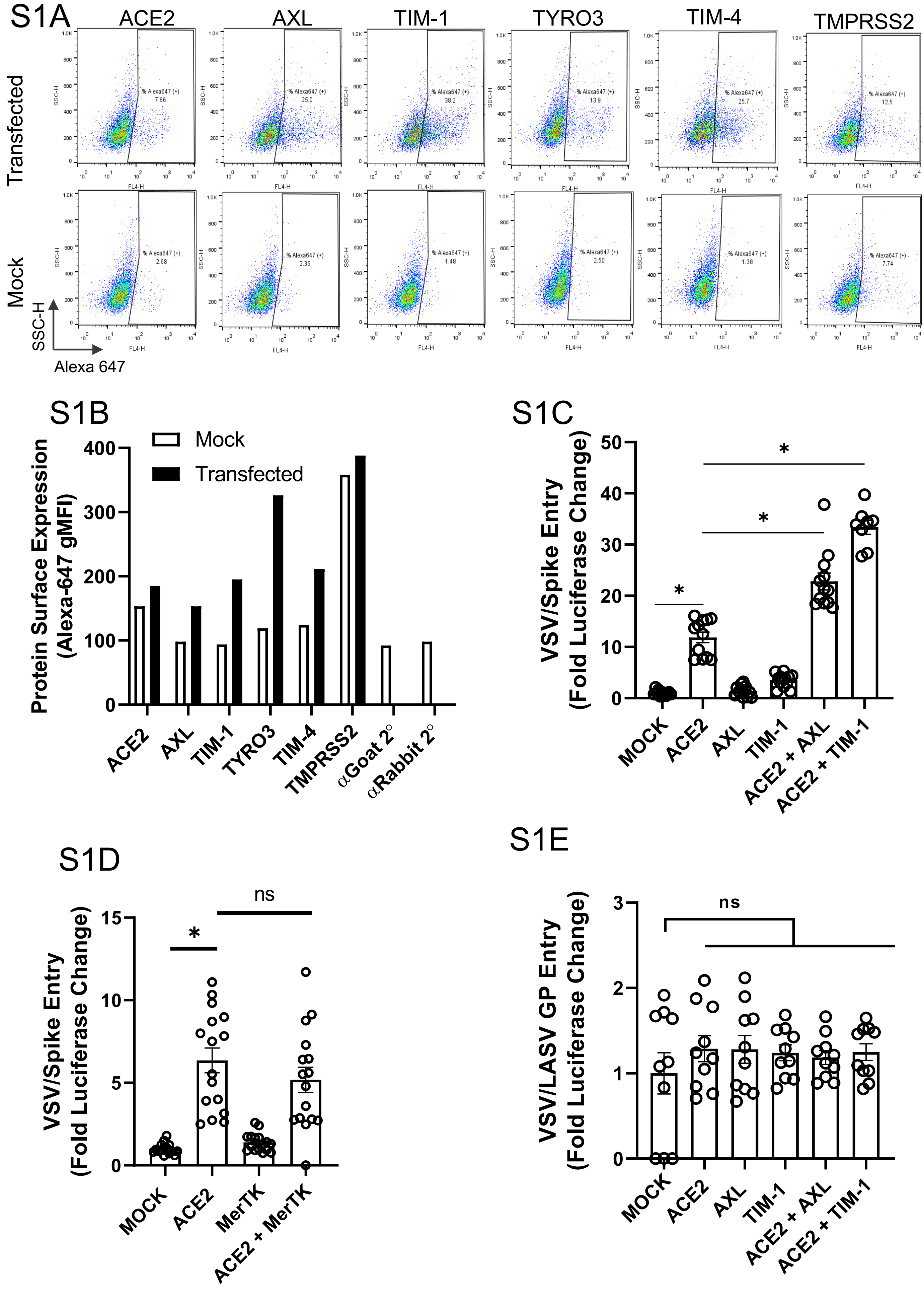
S1A
ACE2
AXL
TIM-1
TYRO3
TIM-4
TMPRSS2
Transfected
Mock
SSC-H
Alexa 647
S1B
Mock
Transfected
Protein Surface Expression (Alexa-647 gMFI)
ACE2
AXL
TIM-1
TYRO3
TIM-4
TMPRSS2
αGoat 2°
αRabbit 2°
S1C
VSV/Spike Entry (Fold Luciferase Change)
MOCK
ACE2
AXL
TIM-1
ACE2 + AXL
ACE2 + TIM-1
S1D
VSV/Spike Entry (Fold Luciferase Change)
ns
MOCK
ACE2
MerTK
ACE2 + MerTK
S1E
VSV/LASV GP Entry (Fold Luciferase Change)
ns
MOCK
ACE2
AXL
TIM-1
ACE2 + AXL
ACE2 + TIM-1

S1 Fig: PS receptors synergize with ACE2, enhancing SARS-CoV-2 infection of HEK 293T cells. S1A) Representative surface staining of receptors transfected into cells. **S1B**) Surface expression (MFI) of proteins in mock transfected (empty vector) and transfected HEK 293T at 48 hours after transfection. Background fluorescence is shown for secondary antibodies used in experiment (α-goat or rabbit secondaries). **S1C**) HEK 293T cells, transfected PS receptors as noted with or without 250 ng of ACE2 were transduced with rVSV/Spike. Transduction was assessed 24 hours later via luminescence. **S1D**) Expression of MerTK did not affect rVSV/Spike transduction in the presence of 250 ng of transfected ACE2 plasmid. **S1E**) Expression of ACE2, TIM-1 or AXL did not enhance infection of VSV-luciferase/Lassa virus GP pseudovirions. HEK 293T cells were transfected with PS receptor plasmids and 50 ng of ACE2 and infected 48 hours later. Panels **S1C**, **S1D**, and **S1E** are shown as fold change of luciferase activity in cell lysates relative to mock transfected lysates that were set to a value of 1.

Data shown are pooled from at least three independent experiments (**S1C**, **S1D**, **S1E**). Data represented as means ± SEM. One-Way ANOVA with multiple comparisons (**S1C**, **S1E**), Student's t-test (**S1D**); asterisks represent $p < 0.05$.
