# Supplementary material for: Phosphatidylserine receptors enhance SARS-CoV-2 infection
Source: PLoS Pathog. 2021 Nov 19;17(11):e1009743. doi: 10.1371/journal.ppat.1009743 (PMC8641883; doi:10.1371/journal.ppat.1009743)
Supplement: S2 Fig — A) PS liposomes compete with PS on purified virions for binding to bavituximab. B) TIM-1 mutant ND115DN is highly expressed after plasmid transfection in HEK 293T cells. C) AXL surface expression in transfected HEK 293T cells. D) Purified Spike ectodomain-Fc and NTD-Fc are detected by an NTD monoclonal antibody by ELISA. E) All Spike-Fc proteins bind and are detected at equivalent levels of ELISA plates. Data represented as means ± SEM. Two-Way ANOVA with multiple comparisons (A), One-Way ANOVA with multiple comparisons (B); asterisks represent p < 0.05. (PDF) [file ppat.1009743.s002.pdf]

S2 Fig

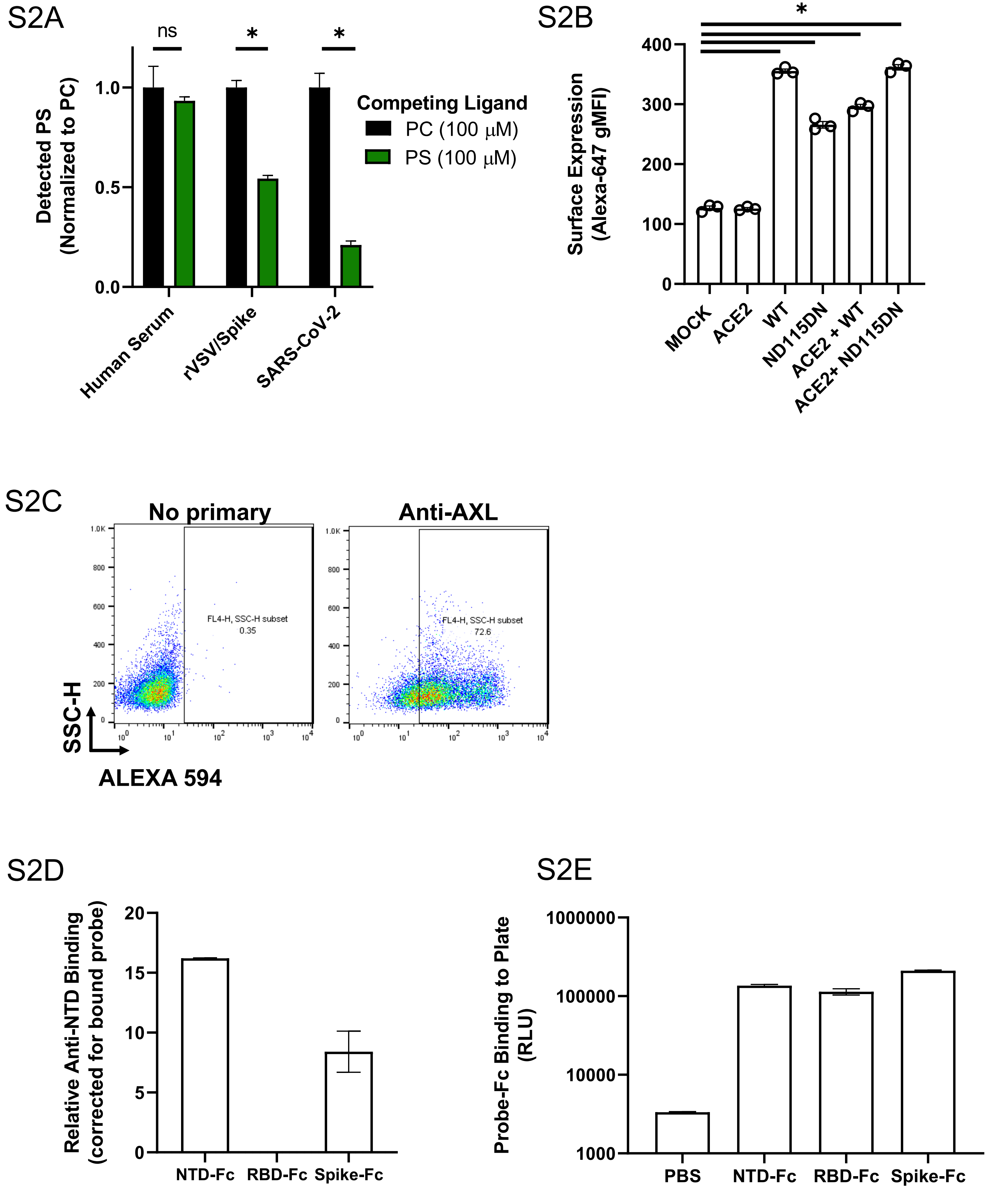
S2A
ns
*
*
Detected PS (Normalized to PC)
1.0
0.5
0.0
Competing Ligand
PC (100 μM)
PS (100 μM)
Human Serum
rVSV/Spike
SARS-CoV-2
S2B
*
Surface Expression (Alexa-647 gMFI)
400
300
200
100
0
MOCK
ACE2
WT
ND115DN
ACE2 + WT
ACE2+ ND115DN
S2C
No primary
Anti-AXL
FL4-H, SSC-H subset
0.35
FL4-H, SSC-H subset
72.6
SSC-H
ALEXA 594
S2D
Relative Anti-NTD Binding (corrected for bound probe)
20
15
10
5
0
NTD-Fc
RBD-Fc
Spike-Fc
S2E
Probe-Fc Binding to Plate (RLU)
1000000
100000
10000
1000
PBS
NTD-Fc
RBD-Fc
Spike-Fc

S2 Fig: PS receptors interact with SARS-CoV-2 by binding to PS.
S2A) PS liposomes compete with PS on purified virions for binding to bavituximab. **S2B**) TIM-1 mutant ND115DN is highly expressed after plasmid transfection in HEK 293T cells. **S2C**) AXL surface expression in transfected HEK 293T cells. **S2D**) Purified Spike ectodomain-Fc and NTD-Fc are detected by an NTD monoclonal antibody by ELISA. **S2E**) All Spike-Fc proteins bind and are detected at equivalent levels of ELISA plates.

Data represented as means ± SEM. Two-Way ANOVA with multiple comparisons (**S2A**), One-Way ANOVA with multiple comparisons (**S2B**); asterisks represent $p < 0.05$.
