# Supplementary material for: Phosphatidylserine receptors enhance SARS-CoV-2 infection
Source: PLoS Pathog. 2021 Nov 19;17(11):e1009743. doi: 10.1371/journal.ppat.1009743 (PMC8641883; doi:10.1371/journal.ppat.1009743)
Supplement: S3 Fig — ATPLite cytotoxicity assay in human lung cells, H1650, 24 hours following treatment with E64. Data are represented as means +/- SEM. (PDF) [file ppat.1009743.s003.pdf]

S3 Fig

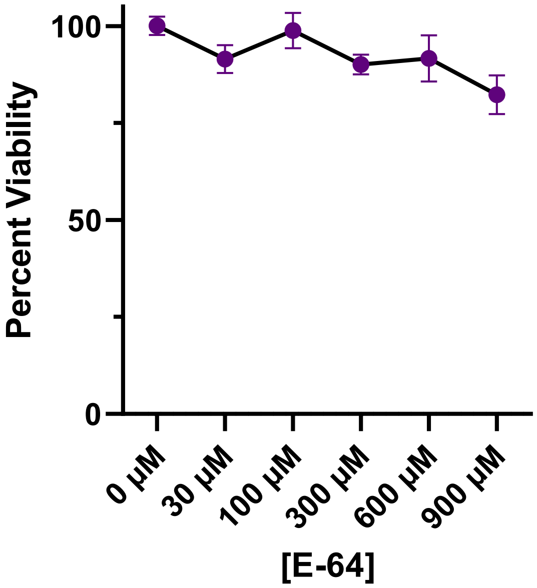

Percent Viability
100
50
0
0 µM
30 µM
100 µM
300 µM
600 µM
900 µM
[E-64]

S3 Fig: The route of SARS-CoV-2 entry is altered by TMPRSS2 expression. ATPLite cytotoxicity assay in human lung cells, H1650, 24 hours following treatment with E64. Data are represented as means ± SEM.
