# Supplementary material for: Phosphatidylserine receptors enhance SARS-CoV-2 infection
Source: PLoS Pathog. 2021 Nov 19;17(11):e1009743. doi: 10.1371/journal.ppat.1009743 (PMC8641883; doi:10.1371/journal.ppat.1009743)
Supplement: S4 Fig — A) ACE2, AXL and TIM-1 surface expression MFI in Vero E6 cells, as assessed by flow cytometry. Background fluorescence is shown for secondary antibodies used in experiment. B) Cell surface versus intracellular ACE2 expression in Vero E6 cells. Cells were lifted, permeabilized as noted, and stained with anti-ACE2 unconjugated primary antibodies and Alexa 647 secondaries and analyzed by flow cytometry. C) Bemcentinib toxicity 24 hours after treatment was measured by ATPlite assay in H1650 cell line. D) VSV/Spike entry was measured by flow cytometry 24 hours after virus challenge of Vero E6 cells treated with bemcentinib. E) Vero E6 were treated with ARD5 (anti-human TIM-1 blocking antibody) 1 hour before infection with rVSV/Spike or rVSV/EBOV-GP (MOI = 0.01). Viral load was measured 24 hpi by RT-qPCR and presented normalized to the highest MOI for each rVSV. F) Plot profiles of ACE2 and AXL intensity are shown from STED micrographs in Fig 4F, representing signal intensity along the yellow lines in the merged panels. Data in A, C, D and E are shown as means ± SEM. Multiple t-tests were performed in C and Student’s t-test was performed in D and E; asterisks represent p < 0.05. (PDF) [file ppat.1009743.s004.pdf]

S4 Fig

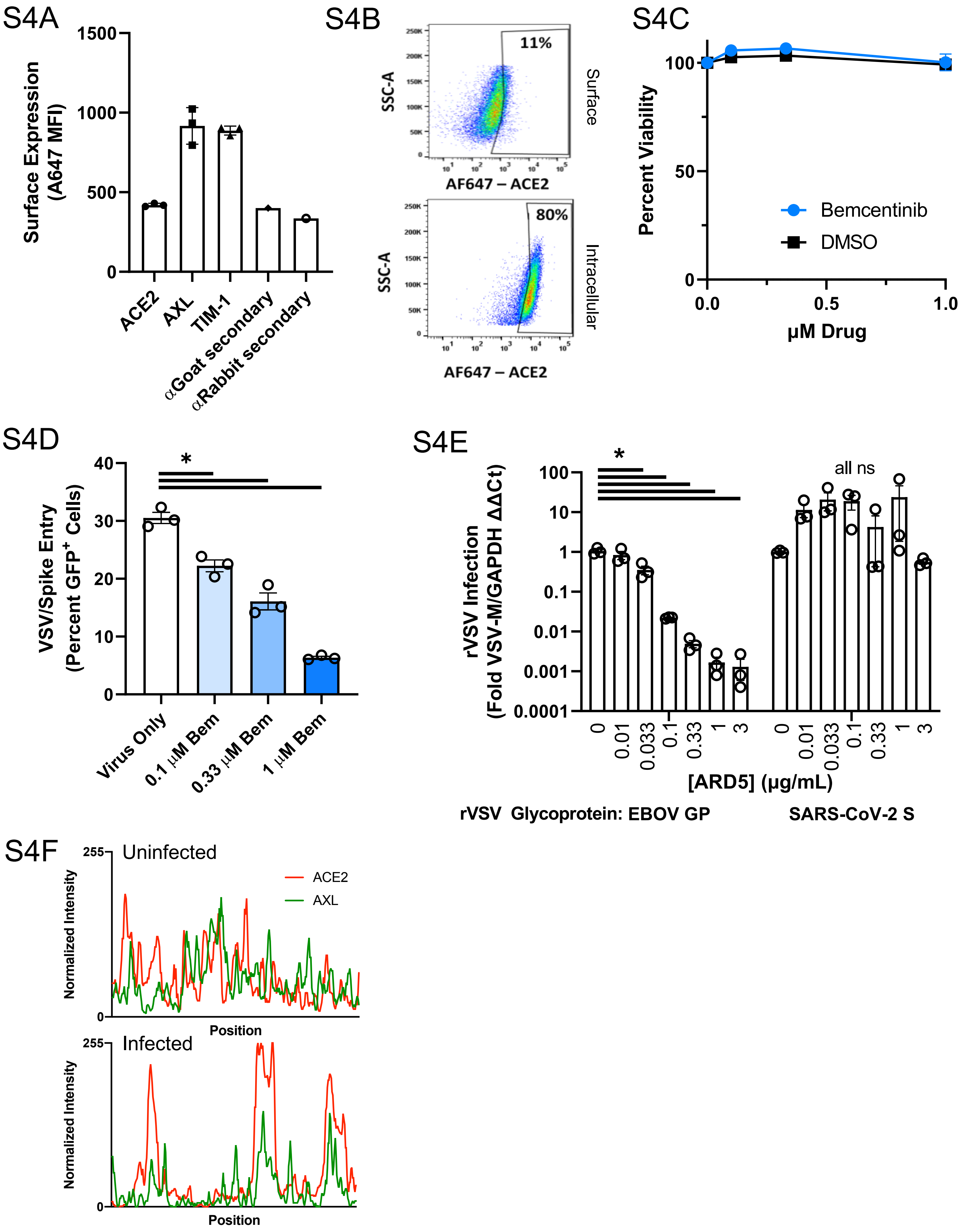

S4A
Surface Expression (A647 MFI)
1500
1000
500
0
ACE2
AXL
TIM-1
αGoat secondary
αRabbit secondary
S4B
11%
SSC-A
AF647 – ACE2
Surface
80%
SSC-A
AF647 – ACE2
Intracellular
S4C
Percent Viability
100
50
0
0.0
0.5
1.0
μM Drug
Bemcentinib
DMSO
S4D
VSV/Spike Entry (Percent GFP⁺ Cells)
40
30
20
10
0
*
Virus Only
0.1 μM Bem
0.33 μM Bem
1 μM Bem
S4E
rVSV Infection (Fold VSV-M/GAPDH ΔΔCt)
100
10
1
0.1
0.01
0.001
0.0001
*
all ns
0
0.01
0.033
0.1
0.33
1
3
[ARD5] (μg/mL)
rVSV Glycoprotein: EBOV GP
SARS-CoV-2 S
S4F
Uninfected
255
0
Normalized Intensity
ACE2
AXL
Position
Infected
255
0
Normalized Intensity
Position

S4 Fig: AXL has a prominent role in SARS-CoV-2 entry in Vero E6 cells. S4A) ACE2, AXL and TIM-1 surface expression MFI in Vero E6 cells, as assessed by flow cytometry. Background fluorescence is shown for secondary antibodies used in experiment. **S4B**) Cell surface versus intracellular ACE2 expression in Vero E6 cells. Cells were lifted, permeabilized as noted, and stained with anti-ACE2 unconjugated primary antibodies and Alexa 647 secondaries and analyzed by flow cytometry. **S4C**) Bemcentinib toxicity 24 hours after treatment was measured by ATPlite assay in H1650 cell line. **S4D**) VSV/Spike entry was measured by flow cytometry 24 hours after virus challenge of Vero E6 cells treated with bemcentinib. **S4E**) Vero E6 were treated with ARD5 (anti-human TIM-1 blocking antibody) 1 hour before infection with rVSV/Spike or rVSV/EBOV-GP (MOI = 0.01). Viral load was measured 24 hpi by RT-qPCR and presented normalized to the highest MOI for each rVSV. **S4F**) Plot profiles of ACE2 and AXL intensity are shown from STED micrographs in Fig 4F, representing signal intensity along the yellow lines in the merged panels. Data (**S4A**, **S4C**, **S4D**, **S4E**) are shown as means ± SEM. Multiple t-tests were performed in **S4C** and Student's t-test was performed in (**S4D**, **S4E**); asterisks represent $p < 0.05$.
