# Supplementary material for: Phosphatidylserine receptors enhance SARS-CoV-2 infection
Source: PLoS Pathog. 2021 Nov 19;17(11):e1009743. doi: 10.1371/journal.ppat.1009743 (PMC8641883; doi:10.1371/journal.ppat.1009743)
Supplement: S5 Fig — A) Human lung cell lines used in these studies were stained for cell surface ACE2, AXL, TIM-1, and TMPRSS2 protein, and expression was quantified by flow cytometry. Shown are flow cytometry histograms depicting target surface staining (black line) and secondary only background (gray shade). B) H1650 cells were treated with 1 μM of bemcentinib and infected with one of three different variants of SARS-CoV-2: WA-1; B.1.1.7 or B.1.351 (MOI = 0.5 for all variants). RNA was isolated at 24 hpi and assessed for virus load. C) PS liposomes do not inhibit SARS-CoV-2 infection in Calu-3 cells. Cells were pretreated with liposomes at indicated doses, infected with SARS-CoV-2, and viral load was assessed 24 hpi. D) H1650 cells were infected with SARS-CoV-2 (MOI = 0.5) after treatment with the indicated concentration of camostat for 1 hour. Viral loads 24hpi were measured by RT-qPCR. E) Cell surface and intracellular staining of ACE2 is shown in multiple cell lines. These data are shown as frequency positive cells. Data represented as means ± SEM. Data are representative of 3 independent experiments (B). Student’s t-test (B, C, E); asterisks represent p < 0.05. (PDF) [file ppat.1009743.s005.pdf]

S5 Fig

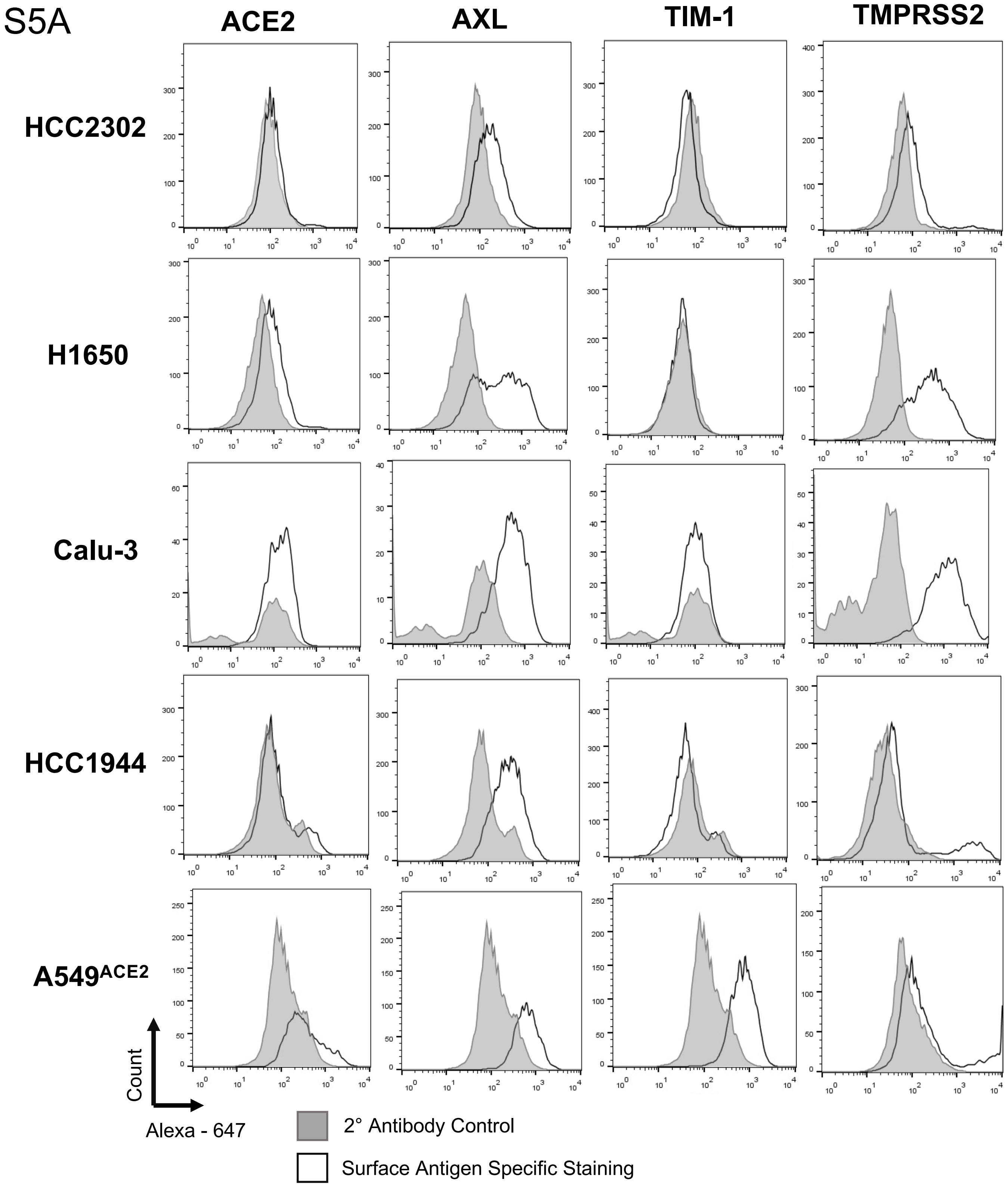
S5A
ACE2
AXL
TIM-1
TMPRSS2
HCC2302
H1650
Calu-3
HCC1944
A549^ACE2
Count
Alexa - 647
2° Antibody Control
Surface Antigen Specific Staining

S5 Fig (continued)

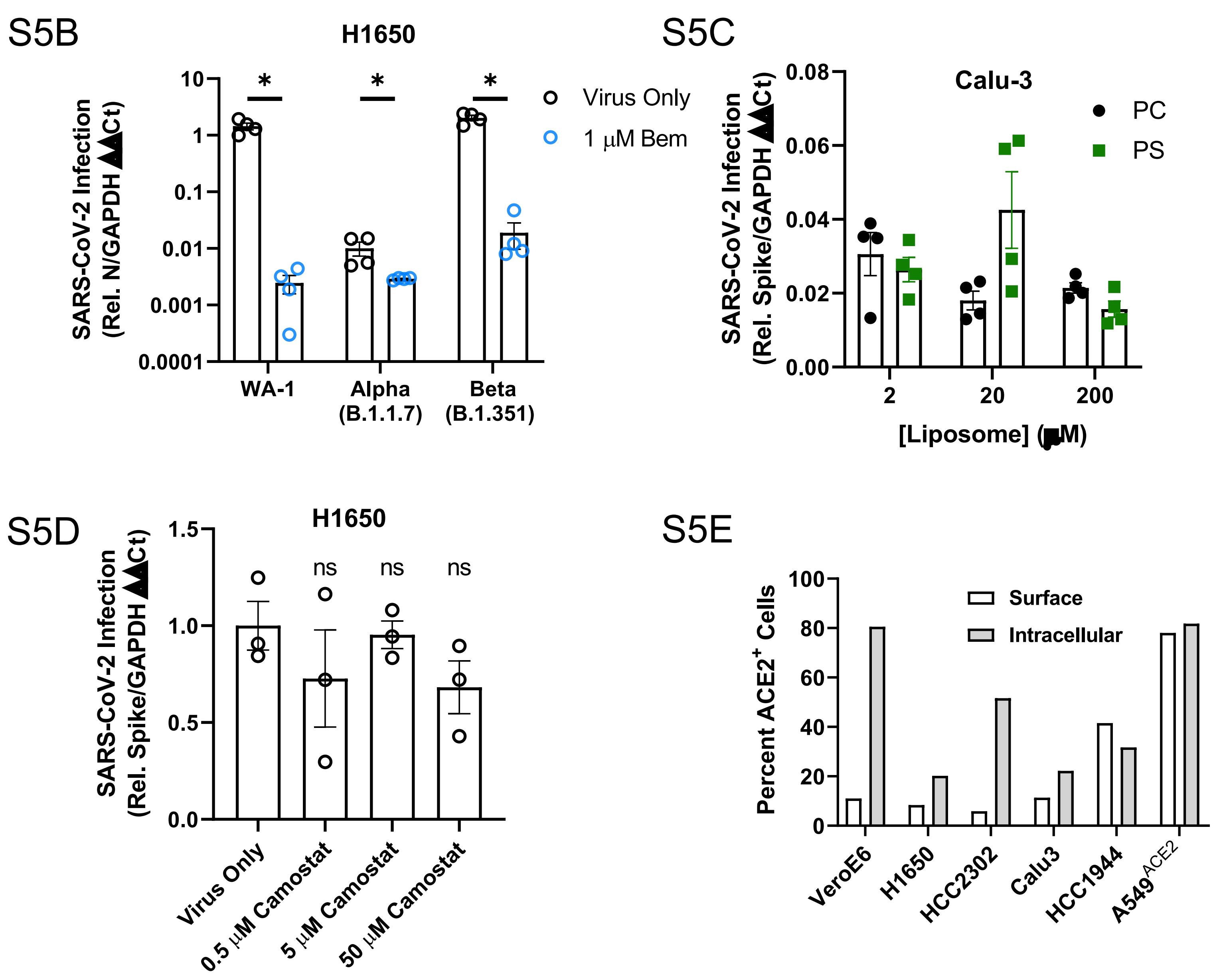

S5 Fig: AXL inhibition reduces SARS-CoV-2 infection in human lung cells. S5A) Human lung cell lines used in these studies were stained for cell surface ACE2, AXL, TIM-1, and TMPRSS2 protein, and expression was quantified by flow cytometry. Shown are flow cytometry histograms depicting target surface staining (black line) and secondary only background (gray shade). **S5B**) H1650 cells were treated with 1 εM of bemcentinib and infected with one of three different variants of SARS-CoV-2: WA-1; B.1.1.7 or B.1.351 (MOI=0.5 for all variants). RNA was isolated at 24 hpi and assessed for virus load. **S5C**) PS liposomes do not inhibit SARS-CoV-2 infection in Calu-3 cells. Cells were pretreated with liposomes at indicated doses, infected with SARS-CoV-2, and viral load was assessed 24 hpi. **S5D**) H1650 cells were infected with SARS-CoV-2 (MOI = 0.5) after treatment with the indicated concentration of camostat for 1 hour. Viral loads 24hpi were measured by RT-qPCR. **S5E**) Cell surface and intracellular staining of ACE2 is shown in multiple cell lines. These data are shown as frequency positive cells. Data represented as means Ñ SEM. Data are representative of 3 independent experiments (**S5B**). Studentõs t-test (**S5B**, **S5C**, **S5E**); asterisks represent $p < 0.05$.
