# Supplementary material for: Phosphatidylserine receptors enhance SARS-CoV-2 infection
Source: PLoS Pathog. 2021 Nov 19;17(11):e1009743. doi: 10.1371/journal.ppat.1009743 (PMC8641883; doi:10.1371/journal.ppat.1009743)
Supplement: S6 Fig — A) Knock down of AXL (AXLlow) in H1650 and HCC2302 cells were generated by lentiviral transduction of Cas9 and gRNA targeting AXL, followed by selection (no clonal isolation). These polyclonal cells are designated “AXLlow”. Shown are flow cytometry histograms depicting AXL surface staining (black) and secondary only background (gray), demonstrating clear reduction of AXL expression in bulk populations of cells. B) H1650 AXLlow and H1650 Cas9 (parental) lines were challenged with SARS-CoV-2 at indicated MOIs for 24 hpi and viral loads assessed by RT-qPCR. C) H1650 parental and AXLlow lines were treated with indicated concentration of bemcentinib and challenged with SARS-CoV-2 (MOI = 0.5) and viral loads determine by RT-qPCR 24 hpi. D-E) As in B-C with HCC2302 cells. Data are pooled from at least 3 independent experiments (B, C, D, E) or are representative of at least 3 experiments (A). Data represented as means ± SEM. Multiple t-tests; asterisks represent p < 0.05. (PDF) [file ppat.1009743.s006.pdf]

S6 Fig

S6A

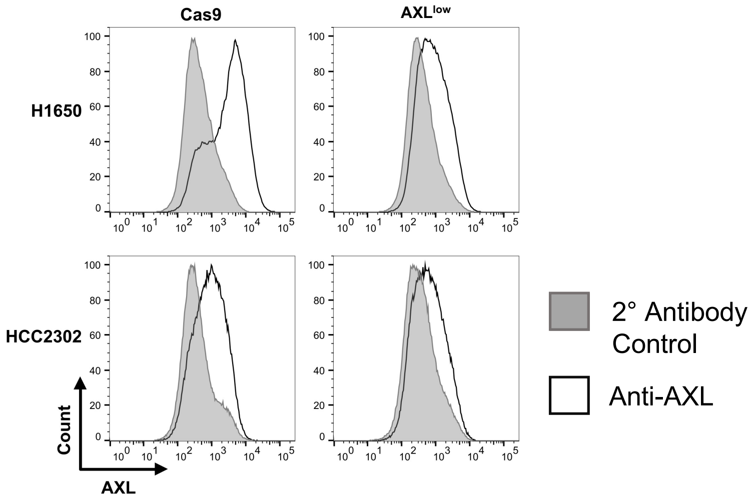

S6B

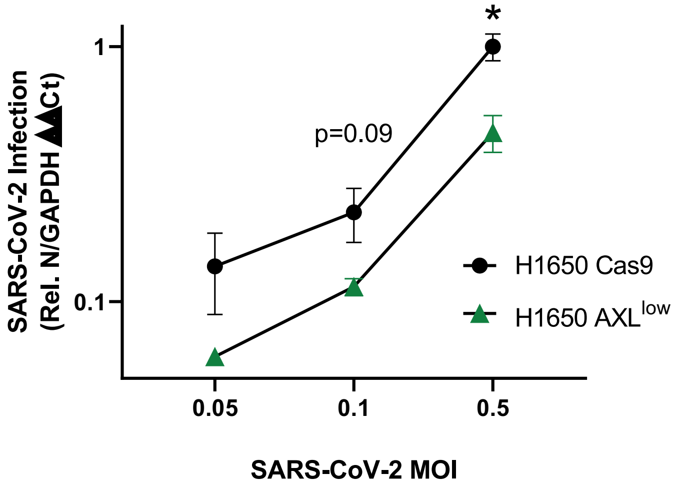

S6C

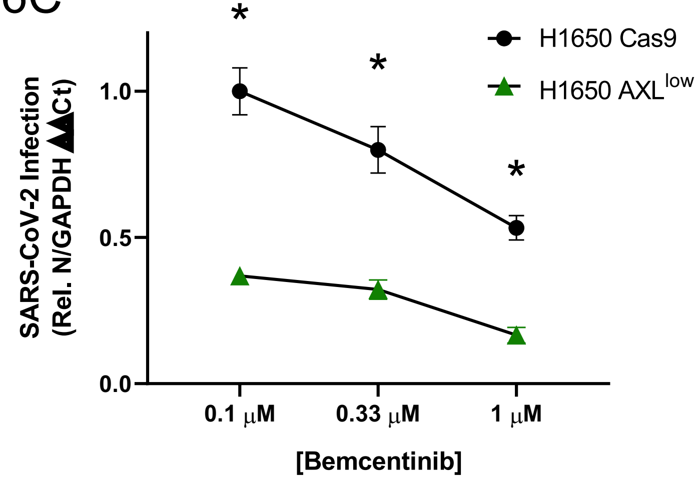

S6D

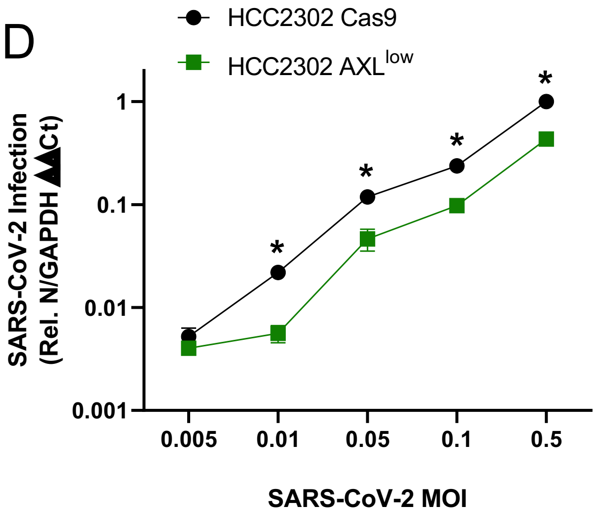

S6E

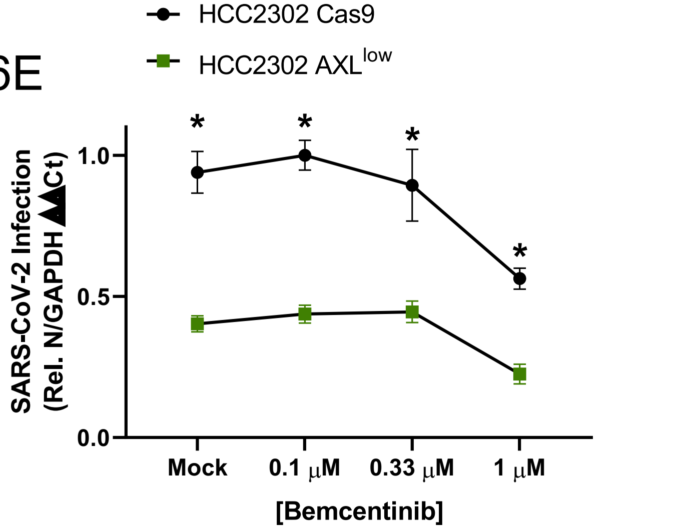

S6 Fig: AXL knockout reduces viral loads and ablates bemcentinib inhibition. S6A) Knock down of AXL (AXL^{low}) in H1650 and HCC2302 cells were generated by lentiviral transduction of Cas9 and gRNA targeting AXL, followed by selection (no clonal isolation). These polyclonal cells are designated "AXL^{low}". Shown are flow cytometry histograms depicting AXL surface staining (black) and secondary only background (gray), demonstrating clear reduction of AXL expression in bulk populations of cells. **S6B**) H1650 AXL^{low} and H1650 Cas9 (parental) lines were challenged with SARS-CoV-2 at indicated MOIs for 24 hpi and viral loads assessed by RT-qPCR. **S6C**) H1650 parental and AXL^{low} lines were treated with indicated concentration of bemcentinib and challenged with SARS-CoV-2 (MOI = 0.5) and viral loads determine by RT-qPCR 24 hpi. **S6D** and **S6E**) As in **S6B** and **S6C** with HCC2302 cells.

Data are pooled from at least 3 independent experiments (**S6B**, **S6C**, **S6D**, **S6E**) or are representative of at least 3 experiments (**S6A**). Data represented as means ± SEM. Multiple t-tests; asterisks represent $p < 0.05$.
