# Supplementary material for: Phosphatidylserine receptors enhance SARS-CoV-2 infection
Source: PLoS Pathog. 2021 Nov 19;17(11):e1009743. doi: 10.1371/journal.ppat.1009743 (PMC8641883; doi:10.1371/journal.ppat.1009743)
Supplement: S7 Fig — A) Bone marrow derived macrophages from C57bl6/J mice were treated as indicated and challenged with MHV (strain A59) at the indicated MOI. Viral loads were assessed 24 hpi by RT-qPCR. B) BMDMs were treated with indicated concentrations of bemcentinib for 1 hour, infected with MHV (MOI = 0.001) for 24 hours and viral load assessed by RT-qPCR. C) As in B, MHV infection of murine peritoneal macrophages (MOI = 0.001) treated with indicated concentrations of bemcentinib. D) Peritoneal macrophages from WT and AXLlacz mice were infected with MHV at multiple MOIs, and viral load was quantified 24 hpi. E) VSV/Spike pseudovirions were incubated with Vero E6 cells in the presence of the indicated compounds, and infection was assessed for luciferase activity 48 hours later. Data shown are 2 pooled (D, E) or representative of 3 independent (A, B, C) experiments. Data represented as means ± SEM. Student’s t-test; asterisks represent p < 0.05. (PDF) [file ppat.1009743.s007.pdf]

S7 Fig

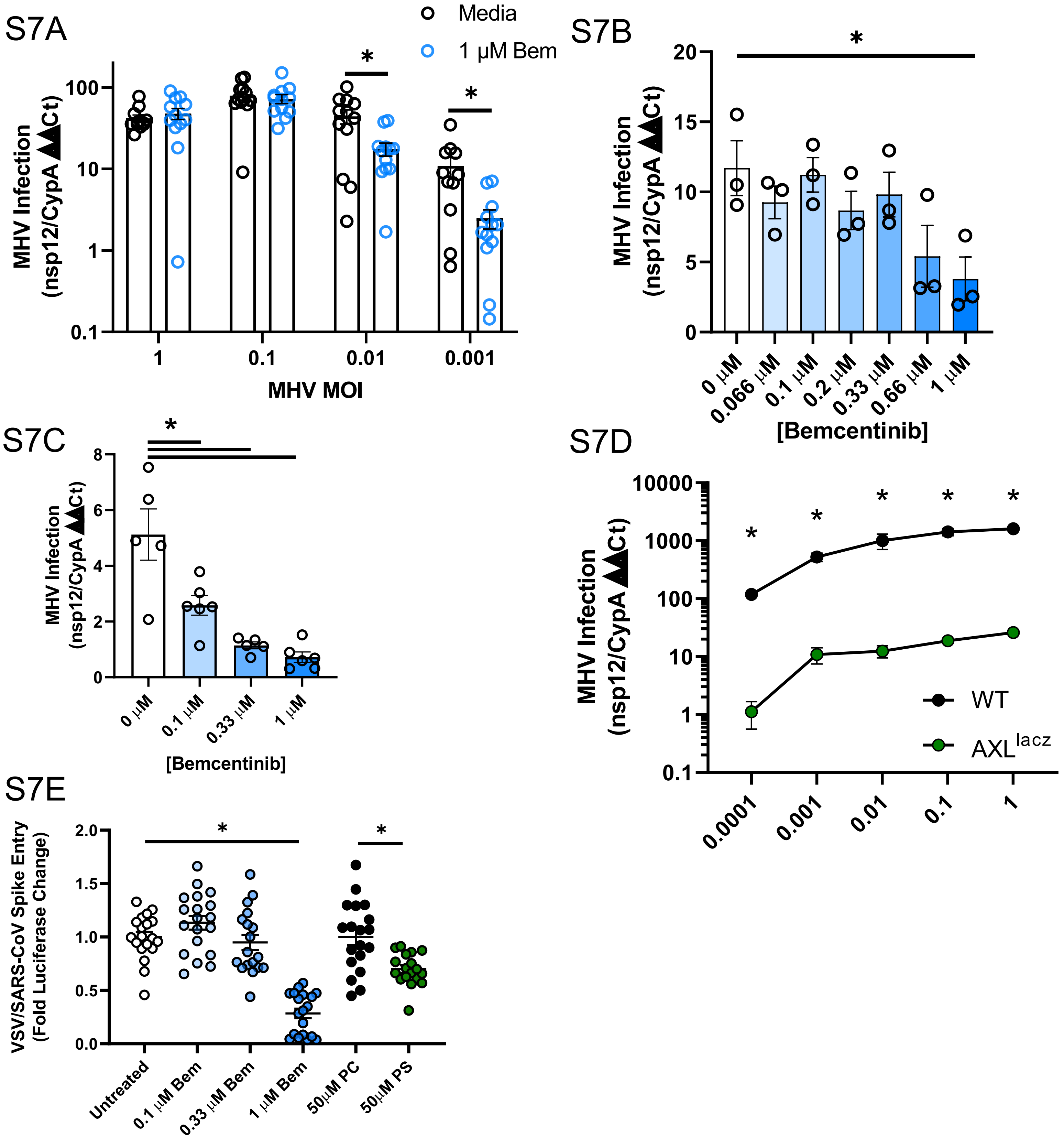
S7A
Media
1 μM Bem
MHV Infection
(nsp12/CypA ΔΔCt)
100
10
1
0.1
*
*
1
0.1
0.01
0.001
MHV MOI
S7B
*
20
15
10
5
0
0 μM
0.066 μM
0.1 μM
0.2 μM
0.33 μM
0.66 μM
1 μM
[Bemcentinib]
S7C
*
8
6
4
2
0
0 μM
0.1 μM
0.33 μM
1 μM
[Bemcentinib]
S7D
10000
1000
100
10
1
0.1
*
*
*
*
*
WT
AXL^{lacz}
0.0001
0.001
0.01
0.1
1
S7E
VSV/SARS-CoV Spike Entry
(Fold Luciferase Change)
2.0
1.5
1.0
0.5
0.0
*
*
Untreated
0.1 μM Bem
0.33 μM Bem
1 μM Bem
50μM PC
50μM PS

S7 Fig: S7A) Bone marrow derived macrophages from C57bl6/J mice were treated as indicated and challenged with MHV (strain A59) at the indicated MOI. Viral loads were assessed 24 hpi by RT-qPCR. **S7B**) BMDMs were treated with indicated concentrations of bemcentinib for 1 hour, infected with MHV (MOI = 0.001) for 24 hours and viral load assessed by RT-qPCR. **S7C**) As in B, MHV infection of murine peritoneal macrophages (MOI =0.001) treated with indicated concentrations of bemcentinib. **S7D**) Peritoneal macrophages from WT and AXL^{lacz} mice were infected with MHV at multiple MOIs, and viral load was quantified 24 hpi. **S7E**) VSV/Spike pseudovirions were incubated with Vero E6 cells in the presence of the indicated compounds, and infection was assessed for luciferase activity 48 hours later.
Data shown are 2 pooled (**S7D**, **S7E**) or representative of 3 independent (**S7A**, **S7B**, **S7C**) experiments. Data represented as means ± SEM. Student’s t-test; asterisks represent $p < 0.05$.
