# Supplementary material for: Phosphatidylserine receptors enhance SARS-CoV-2 infection
Source: PLoS Pathog. 2021 Nov 19;17(11):e1009743. doi: 10.1371/journal.ppat.1009743 (PMC8641883; doi:10.1371/journal.ppat.1009743)
Supplement: Attachment — Submitted filename: Response to reviewers 211008_HVE comments.docx [file ppat.1009743.s008.docx]

Response to reviewers:

Attached is a revised version of manuscript PPATHOGENS-D-21-01321. We have made a number of changes to the manuscript that include those requested by the reviewers. We would like to thank the reviewers for their thoughtful comments. We believe that we have addressed the concerns of the reviewers and details are noted below. The reviewers’ comments are in black New Times Roman, whereas our disposition of the comments is shown in blue Arial. The revised manuscript is much improved. We believe this manuscript is now ready for publication.

Additional changes to the manuscript that were not in response to the reviewers:

1. A shortened title

2. Added an additional author

3. Added an additional keyword

Reviewer's Responses to Questions

**Part I - Summary**
Please use this section to discuss strengths/weaknesses of study, novelty/significance, general execution and scholarship.

Reviewer #1: This submission provides indirect but compelling evidence that TIM and TAM receptors (or TAM adaptors) bind phosphatidyl serine (PS) on SARS-CoV-2 (SARS2) pseudoviruses and authentic viruses, and that these TIM/TAM:PS interactions facilitate virus-cell binding and ACE2-dependent virus-cell entry. The findings help to clarify the field, particularly because a prior report claims that one TAM (called AXL) binds directly to SARS spike protein domains. This submission argues against this TAM-spike binding and makes the more conventional claim of PS binding, consistent with abundant prior data on TIM/TAM interactions with PS on several diverse enveloped virus membranes. Furthermore, this submission makes the sensible claims that TIM/TAM molecules are only recognized as assisting SARS2 entry when ACE2 levels are low, and that ACE2 is still required even when TIM/TAM are abundant. The data supporting these claims are generally strong, although the levels at which TIM/TAM receptors support SARS2 entry are often fairly modest. Physiological relevance of TIM/TAM in vivo was not addressed. There are also some puzzling findings in the report (for example, TIMs support SARS2 entry in some cell types while TAM (AXL) does so in others, even though both TIMs and TAMs are all concurrently available to the virus). Hence some findings are left unexplained. However, given that identifying and characterizing SARS2 susceptibility factors are valuable in understanding the SARS2 pandemic, the report appears to be a worthy contribution to the field.

Reviewer #2: In this manuscript by Bohan and colleagues, the role of phosphatidylserine (PS) receptor in SARS-CoV-2 entry is investigated. In this study, the authors first show that, while not sufficient for infection, expression of PS receptor Axl, TIM-1, and TIM-4, but not TYRO3 or MerTK, increases SARS-CoV-2 infection of 293T cells expressing low levels of Ace2 or a wider range of level of expression of Ace2 for TIM-1. This enhancement of entry correlated with an increase in virion attachment to cells in the presence of Axl and TIM-1. The authors then show that liposomes containing PS block the enhancement of infection conferred by the expression of the PS receptor, and, using a TIM-1 mutant, that the ability of TIM-1 to bind PS is essential for its function as an entry factor. Given that the authors did not detect an interaction between Axl and SARS-CoV-2 S or the S N-terminal domain, they conclude that the enhancement of infection by the PS receptors is via interaction with exposed PS in the viral envelope. The authors then investigated whether the PS receptor-dependent enhancement of infection is restricted to one particular route of entry (surface vs endosomal). As others, they showed that co-expression of TMPRSS2 with Ace2 redirect entry from an endosomal route that is E64 sensitive to a surface entry that is E64 insensitive. The additional expression of PS receptor did not modify the entry route which was dictated by TMPRSS2 presence or absence. The presence of PS did not seem to enhance infection in the context of TMPRSS2 co-expression in 293T-Ace2, although the authors did not comment on this or explore this further. The authors then study the roles of these receptors in Vero cells, which endogenously express Ace2, Axl, and TIM-1. In these cells, PS liposomes and a specific Axl kinase inhibitor reduced infection, but a TIM-1 antibody had no effect, suggesting potential differences in PS receptor usage in different cell lines. In addition, the authors found that upon infection, colocalization of Axl and Ace2 is increased and localized in intracellular vesicles, providing some insight into a potential (additional?) mechanism of action of the PS receptors. The authors then looked at the contribution of Axl, using their kinase inhibitor, to infection of various lung cell lines. The effect of the Axl inhibitor correlated with a sensitivity to the cysteine protease inhibitor E64. Using CRISPR/Cas9, the authors generated Axl KO and found that viral growth was dramatically reduced, and residual infection was insensitive to the Axl kinase inhibitor. Finally, they show that infection by another coronavirus, MHV, is also sensitive to the Axl kinase inhibitor, suggesting a wide PS receptor usage by coronaviruses.

Overall, the study is well-conducted with the use of controls and multiple tools to dissect the role of PS receptors in SARS-CoV-2 infection. The conclusions are generally well supported although there is some overinterpretation with regards to the potential mode(s) of interaction with PS receptors. In addition, a better characterization of the roles of PS receptors in the enhancement of infection (attachment, internalization, and or trafficking to Ace2), and whether this only applies to endosomal entry would strengthen this study.

**Part II – Major Issues: Key Experiments Required for Acceptance**
Reviewer #1: 1. The AXL inhibitor bemcentinib (bem) figures prominently in the study and bem is used frequently to assess AXL function in SARS2 entry. At least in Vero cells, data appear to suggest bem is pleiotropic and it is not so clear that its antiviral activity is due entirely to AXL inhibition. This is because the bem drug is far more antiviral than PS liposomes, at least in comparing Figs 4B and 4C.

Thank you for these comments. Yes, bemcentinib does inhibit SARS-CoV-2 more robustly in our hands than PS liposomes. Interestingly, this is particularly true in Vero E6 cells – and to a less extent in the human lung lines that we study. This may be due to additional antiviral effects of bemcentinib (see below) or due to the inefficiency of PS liposome competition or a combination of both.

Bemcentinib has been shown to be a highly specific inhibitor of the kinase activity of AXL (Holland et al (2010) Cancer Research. DOI: 10.1158/0008-5472.CAN-09-2997), but the activities of AXL that bemcentinib blocks are wide ranging. Gas6/AXL complexes mediate not only PS binding and internalization, these complexes also stimulate cell division, angiogenesis, cell migration and metastasis of cancer cells (Yan, et al. Cancers DOI: 10.3390/Cancers11111727). Thus, bemcentinib inhibition of all of these AXL characteristics may contribute to the antiviral effects observed with bemcentinib. Certainly, one aspect of this may be the ability of Gas6/AXL complexes to suppress type 1 interferon responses (Rothlin et al (2007) Cell DOI: 10.1016/j.cell.2007.10.034). Thus, bemcentinib inhibition of AXL will stimulate type 1 interferon responses, enhancing innate immune responses and impacting SARS-CoV-2 replication. Again, this is now discussed in the Discussion starting on line 484.

On lines 225-227 it is stated that a broad-spectrum TAM inhibitor is inert, therefore bem doesn’t have off-target effects. This rationale is not understood by this reviewer.

Our apologies. We believe there was a misunderstanding in regard to this sentence; we did not mean to imply that the broad-spectrum TAM inhibitor was inert. That inhibitor did inhibit SARS-CoV-2 infection in a modest, but dose dependent manner. We attempted to say that this finding supports our bemcentinib data. We have now changed the wording of these lines and now say “A broad-spectrum TAM inhibitor, BMS777607, modestly reduced virus infection in a dose dependent manner (**Fig. 4F**), reinforcing our observation that inhibition of TAM family members blocks SARS-CoV-2 infection”.

Also, the results from experiments with the bem drug do not concur. For example, Fig 4C, the bem drug effects 2.5 log10 reductions of SARS2, but in Fig 4D, the same bem drug at same conditions effects only 3-fold reduction. Also in Fig 4E, the drug has modest antiviral activity. What explains this?

We agree that the impact of bemcentinib varies between different cells types tested. Studies shown in Figure 4C are in Vero E6 cells, where bemcentinib robustly inhibits SARS-CoV-2 infection (~2.5 logs of inhibition). Studies shown in the old Fig. 4D are in the human lung cells H1650 and in the revised manuscript are moved to Fig. 5 with the other lung cell data. H1650 cells along with the other human lung lines in Fig. 5 are inhibited by bemcentinib in a nice dose dependent manner, but inhibition is more modest than that observed in Vero E6 cells. The range of bemcentinib in the Fig. 5 studies is between 70-95% - roughly the same range observed in H1650 cells in old Fig. 4D (new Fig. 5F). We now note that effect of bemcentinib on SARS-CoV-2 in human lung cells is more modest than that observed in Vero E6 (line 313-314).

Finally, from Fig. 6, it is concluded that the bem antiviral effect must be due to interference with AXL, because bem is less antiviral in AXL KO cells. But here and in Fig S6C and S6E, there are still bem antiviral activities in AXL KO or AXL-low cells.

We believe the knockout studies support our claim. The H1650 clonal AXL knockout line that does not express detectable AXL is no longer sensitive to bemcentinib (Fig. 6C). In this study, the bemcentinib reduction in virus infection in WT H1650 is ~14-fold and highly statistically significant. We have clarified this relationship by denoting the p-value in the figure comparing infected cells with infected + 1uM bemcentinib cells. In contrast, bemcentinib reduction of SARS-CoV-2 infection in the H1650 knockout line is not statistically significant and is ~2-fold, indicating that when AXL is not present bemcentinib does not mediate statistically significant inhibition of SARS-CoV-2. We acknowledge in the Results (line 355) that bemcentinib does modestly inhibit virus replication in the knockdown cells, but we also point out that AXL expression is not completely abolished in this bulk population of cells (S6A).

Some additional statistical analyses of the bem specific antiviral effects in the different cells should be further considered. This issue of bem specificity seems to matter particularly in the MHV study, because bem is used exclusively to make the claim that MHV entry into cells is promoted by interaction with PS receptors. Might off-target antiviral effects of bem in the BMDMs and peritoneal macs explain the findings in Fig S7? Could other tests, orthogonal to the pharmacologic approach with bem, be used to buttress the claims about MHV using AXL?

We show now in S7D that dose response curve of MHV infection is reduced by ~100-fold in AXL-null murine macrophages compared to WT macrophages. This is independent demonstration of the importance of AXL in MHV infection and bolsters our observations that bemcentinib inhibition of AXL blocks MHV replication. All of the MHV studies are shown as supplemental figures.

2. Findings are presented in challenging ways, for example, in Fig 1, results from experiments with authentic SARS2 are intermingled with results coming from SARS2 (VSV) pseudovirus transductions. Might the data be better separated? Along these lines, in comparing all results from experiments with authentic SARS2 to those with VSV pseudo SARS2, are there significant differences in TIM/TAM support or inhibition by PS liposomes or bem? Any knowledge of different PS levels on VSV vs. authentic SARS2, or any information on Gas6 binding to either of the different virus particles?

Thank you for pointing this out. It is true that particularly for the HEK 293T cell studies, many of the experiments were done with VSV pseudovirions that we term VSV/Spike or recombinant VSV that encodes spike that we call rVSV/Spike. The pseudovirions contain one of two reporter genes (GFP or luciferase), whereas the rVSV/Spike encodes nanoluciferase. In these studies, we do intermingle panels within the same figure showing VSV/Spike, rVSV/Spike and SARS-CoV-2 data since the point(s) being made are demonstrated independently by use of the different viruses. However, we have now tried to clarify which virus is being used in any given panel by details in the Y axis of each panel. We hope that this makes our experiments easier to (line follow.

Data collect and trends observed from studies with VSV pseudovirions or rVSV bearing SARS-CoV-2 spike were found to be similar to those we observed with SARS-CoV-2. As a consequence, all three sets of findings are reported as others in the field have done (Hoffmann et al (2020) Cell DOI: 10.1016/j.cell.2020.02.052; Case, et al (2020) Cell Host and Microbe DOI:10:1016/j.chom.2020.06.021; Zang (2020) PNAS DOI: 10.1073/pnas.2012197117).

We have now measured the presence of PS on both rVSV/Spike and SARS-CoV-2 (new Fig. 2A). PS was readily detectable on the surface of these virions. We used an adenoviral vector (non-enveloped) as a negative control and, at each concentration of virus, PS was significantly higher on both enveloped viruses than on adenovirus. To your point, in these studies the quantity of PS on SARS-CoV-2 consistently trended higher than that observed on rVSV/Spike and this is noted in the Results (line 168). However, additional more in-depth studies would be needed to definitively conclude that PS was more abundant on SARS-CoV-2 particles than on rVSV/Spike particles. Thus, these additional studies are beyond the scope of the current work.

Reviewer #2: While I also favor the hypothesis that the observed enhancement of infection by the PS receptor is via virion associated PS instead of direct interaction with S, I believe that the authors have not provided the evidence needed to conclude that it is the case (lines 328-330). For instance, the reduction in the effect of PS receptors on infection by the addition of PS liposome could be due to competition with virion associated PS, as the authors propose, or on the activation and internalization of the PS receptors which would then be unavailable to the virus regardless of how it interacts, if it interacts, with the PS receptors.

As the reviewer notes below, we do not solely demonstrate that virion PS/PS receptor interactions are occurring by blocking the interaction with PS liposomes. We support the PS liposome data with evidence that the TIM-1 mutant ND115DN that is known to not bind to PS in apoptotic bodies (Santiago, et al Immunity DOI: 10.1016/j.immuni.2007.11.008) and filovirus particles (Moller-Tank et al (2013) J. Virol. DOI: 10.1128/JVI.01025-13) also significantly reduces SARS-CoV-2 infection compared to WT TIM-1. These data independently support our contention that PS on SARS-CoV-2 virions is binding to the PS receptors.

The use of the TIM-1 mutant is elegant, but no surface expression is presented. The authors have used this mutant in other studies, I may have missed it, but I was unable to find surface expression data in those as well.

We now show surface staining of the TIM-1 mutant (see S2B). Surface expression of the mutant is not statistically different than expression of WT TIM-1.

Although the data with purified recombinant soluble proteins are convincing, given that the enhancement of infection and even the increase virus attachment to cells seem to be dependent on Ace2, would it be possible that a first interaction of S with Ace2 would be required for secondary interaction with Axl or TIM-1? I would therefore urge the authors to either provide direct evidence that PS exposed on the viral surface (on SARS-CoV-2, or even on SARS-CoV-2 S-VSV) is required for the enhancement of infection, maybe via annexin V binding and competition, or to tone down their statements.

Thank you for the suggestion. Reviewer #1 also requested these data. As noted above, we have now measured the presence of PS on both rVSV/Spike and SARS-CoV-2 (new Fig. 2A). PS was readily detectable on the surface of these virions and was significantly inhibited by the use of competing PS liposomes (S2A). We used an adenoviral vector (non-enveloped) as a negative control and at each concentration of virus the quantity of PS was significantly higher on the enveloped viruses than on adenovirus. We agree that inclusion of this finding strengthens this manuscript.

One important aspect of the potential enhancement of infection by PS receptors that is underdeveloped in this study is whether this is a mechanism that generally applies to SARS-CoV-2 entry or only one of the potential routes of entry (endosomal in that case). This is crucial to carefully assess given that this may have implications for the in vivo relevance of this mechanism. Based on Fig. 3C and the fact that Axl did not seem to be involved in serine protease-dependent surface entry by SARS-CoV-2 in lung cells (Calu3), one could argue that this mechanism only applies to the endosomal route. Unfortunately, infection of the only other lung cell line that the authors tested that seemed to express TMPRSS2 (although its activity remains to be confirmed) was E64 sensitive, suggesting that entry was via the endosomal route. Since the mechanism of Bemcentinib remains to be determined (third point below), other ways of testing a role for PS receptors would be to study the effect of PS liposomes on infection in Calu3 cells.

We have now performed PS/PC competition studies (see S5C). At a wide range of PS concentrations, PS liposomes were unable to inhibit Calu-3 infection. These findings are consistent with other studies (such as the bemcentinib studies in Fig. 5G) with Calu-3 in the manuscript.

Our data with Calu-3 cells and TMPRSS2 transfected HEK 293T cells are consistent and indicate that **in these cells** E64-dependent entry (and AXL utilization when AXL is present) is not utilized if TMPRSS2 is present. However, our observations with other human lung cell lines (eg, H1650) call into question a model that would conclude that, when TMPRSS2 and ACE2 are present, SARS-CoV-2 always enters at the plasma membrane, making virus entry AXL independent as well as E64 insensitive. Instead, H1650 cells express abundant AXL and TMPRSS2 as well as ACE2 and is sensitive to E64, providing evidence that despite expression of TMPRSS2 an endosomal route of virus entry is used. We also clearly demonstrate that SARS-CoV-2 infection of this line is strongly impacted by knockout of AXL. So, while a clean, simple biological model is appealing, biological data can be complex and not always consistent with a simple model. Clearly there are human lung lines such as H1650 that one would predict would utilize a plasma membrane route of entry, but do not. As RNAseq has been performed on H1650 cells by John Minna’s group, we investigated whether TMPRSS2 is mutated in that cell line; it is not. We have also investigated if AXL is mutated in Calu-3 cells. We have found that there is a heterozygous mutation in the AXL gene of Calu-3 cells (G835V). We are currently investigating the impact of that mutation on Calu-3 uptake of SARS-CoV-2 and the biological activities of AXL, but these findings are beyond the scope of this report. We have made note of these intricacies in the manuscript, starting on line 333. “The paradoxical finding that virus entry into H1650 is sensitive to E64 and bemcentinib despite endogenous TMPRSS2 expression indicates that TMPRSS2-dependent pathways are not always the dominating or default route of SARS-CoV-2 entry and suggests that a more complex balance of events controls which pathway is used.”

Alternatively, Vero-TMPRSS2 could also be tested using the multiple tools the authors have used extensively. While the importance of the endosomal entry route in SARS-CoV-2 infection and pathogenesis remains to be determined, the authors should validate in which context(s) PS receptors contribute to entry and infection.

We now add new data demonstrating that PS receptors by themselves when transfected into HEK 293T cells enhance virus internalization (new Fig. 1E). While this result alone does not provide evidence that PS-dependent internalization is mediated through the endosome, the combination of our other studies suggest that PS receptors are internalizing virus into the endosome. These additional data include: 1) as shown in Fig. 3C, E64 blocks virtually all entry by the combination of ACE2 and PS receptors and 2) neither of the PS receptors by themselves enhance SARS-CoV-2 infection (instead a combination of ACE2 and PS receptors is required. From these datasets, the conclusion we have drawn is that PS receptors mediate/enhance uptake of virus into the endosomal compartment where 1) abundant ACE2 is present and/or 2) factors required for spike processing (and membrane fusion) are available. This route of PS receptor mediated entry is consistent with earlier studies on PS receptors that demonstrate that PS receptors stimulate internalization of other viruses (dengue virus, filoviruses) into the endosomal compartment (Moller-Tank et al (2013) J. Virol. DOI: 10.1128/JVI.01025-13; Meertens, et al. Cell Host and Microbe DOI: 10.1016/j.chom.2021.08.009; Jemielity et al (2013) Plos Pathogens DOI: 10.1371/journal/ppat.1003232).

Finally, the actual mechanism(s) of the PS receptors in entry remain to be clarified. At the beginning of the manuscript, using 293T cells, the authors argue for a role of the PS receptor in attachment and only performed binding assays. A role in enhancing surface binding would indicate that these host factors could be involved in both the surface and endosomal entry routes.

We agree with the reviewer that it is plausible that PS receptors could enhance BOTH an endosomal route of entry and a plasma membrane route of entry (through TMPRSS2 dependent events). However, this is not what we have found. When we tested PS receptors for enhancement of fusion at the plasma membrane in the presence of TMPRSS2 and ACE2, we did not find the AXL or TIM-1 enhanced infection and that the virus entered in a E64 independent manner (Fig. 3C).

As we noted above, we have performed internalization studies with HEK 293T overexpressing PS receptors. We observe that PS receptors facilitate virion internalization, and that removal of PS-binding activity (ND115DN TIM-1) or pharmacological inhibition (AXL + bemcentinib) significantly reduce internalization. We believe these data strengthen our conclusion that PS receptors mediate attachment and internalization, as is consistent with the phenomenon of viral apoptotic mimicry.

Later, using Vero cells, binding assays were performed with PS liposomes and these slightly, but significantly, reduced binding. However, Bemcentinib, which is an inhibitor of Axl and binds and inhibits its intracellular kinase domain and thus is not expected to have an effect on PS binding (therefore virus attachment), blocked SARS-CoV-2 infection. Although, virus binding studies in the presence of Bemcentinib are lacking, given the known mechanism of action of the drug, this would suggest a role in a post-attachment step. The authors then show that Axl may be involved in bringing virus/Axl complexes to intracellular Ace2. However, the authors do not show that Bemcentinib prevents this colocalization.

To address this, we performed virus internalization studies (new Fig. 1 E). We found that AXL stimulated internalization and bemcentinib significantly decreases AXL-dependent internalization.

In the conclusion, the authors state that they provide “evidence that PS receptors enhance SARS-CoV-2 binding to cells and mediate internalization into endosomes”. However, without internalization and trafficking assays that specifically assess PS receptor contributions (in 293T cells expressing the PS receptors and the use of Bemcentinib for the Axl/Ace2 colocalisation studies), the authors’ conclusions are not well supported.

Thank you for your comments. As noted above, we now show rVSV/Spike internalization studies (new Fig. 1E). Expression of ACE2, TIM-1 or AXL enhance virion internalization. In contrast, the TIM-1 ND115DN or AXL in the presence of bemcentinib demonstrated significantly reduced levels of virion internalization, supporting the importance of these PS receptors in virus internalization.

In addition, we have cited work done previously demonstrating that during SARS-CoV infection ACE2 form large cytosolic punctate patterns that colocalize with early endosome protein EEA1 (Wang, HL et al., (2008) Cell Research. Doi: 10.1038/cr.2008.15). In agreement with these previous studies, we demonstrate in our STED experiments, that during infection with SARS-CoV-2 in Vero E6 cells, we also see ACE2 forming large cytosolic punctate structures, consistent with the possibility that ACE2 is collecting in endosomes during SARS-CoV-2 infection. Lastly, we see the additional presence of AXL in these punctate structures during SARS-CoV-2 infection, indicating to us that AXL could be assisting ACE2 in allowing virion entry to endosomes.

**Part III – Minor Issues: Editorial and Data Presentation Modifications**
Please use this section for editorial suggestions as well as relatively minor modifications of existing data that would enhance clarity.

Reviewer #1: 3. High ACE2 levels allow SARS2 entry to take place without much need for TMPRSS2 or PS receptors. This is expected and data are shown in Fig 3. But how are the results “related to effects of soluble ACE2 on entry” (line 175)? Some further explanation is needed here.
This section has been expanded, with regards to the renin angiotensin literature. Overexpression of ACE2 leads to proteolytic cleavage of the transmembrane protein and release of soluble ACE2. This sACE2 retains its ability to bind to SARS-CoV-2 spike, and this thus able to undergo receptor mediated endocytosis via the type I angiotensin or vasopressin V1b receptors, potentially providing an additional mechanism of virion uptake. This remark is not intended to be conclusive, but merely suggests alternative explanation of the data.

4. The findings of abundant intracellular ACE2 (lines 195-196 and Fig S4B) are used to make the point that PS receptors bring SARS2 into ACE2-rich endosomes (lines 229-247). The presentation of S4B and lines 195-196 is out of place and confusing. It should be moved to the relevant part of the results section.

We apologize. Lines 195-196 have been removed, and we believe the remaining reference to S4B in 229-247 is now clear.

Reviewer #2: Line 90: please replace permissive for susceptible.

This has been corrected.

Line 119 and legend of Figure 1, and other places in the manuscript: I believe that the use of “synergize” for the enhancement of infection conferred by the expression of the PS receptors is incorrect. This is a minor point, but since the PS receptors do not allow entry on their own, potentiation would be the correct terminology. I would recommend to the authors to revise the terminology used in the manuscript regarding the enhancement of infection/entry by the PS receptors.

Thank you for your comment. We agree and this correction has been made.

In figure 6E, is the difference between Ace2 and Ace2+TIM-1 statistically significant?

We assume the reviewer meant 1E. This comparison is not significant, and a correction has been made in the text.

Line 792: I am confused by the legend, I am assuming that 48 hpi, is in fact 48 hours post-transfection. In addition, the section of the method describing the binding assays with 293T cells lacks clarity (lines 623-626). I am assuming that the procedure was the same as for the Vero cells except for the incubation being at room temperature, but this should be clearly stated.

Thank you. the legend correction has been made. The methods for the 293T binding assay have been expanded.

Supplemental Fig.2: the labels for panel B are not visible.

We now show this dataset as a new graph (Prism) and the error is now remedied.

A material and method section describing how the liposomes were prepared is missing. In addition, how were the experiments performed with the liposomes is unclear. The reader is left to guess based on the figure legends which seem to indicate that a pre-treatment was performed in some cases (for instance Fig.4A), while it remains unclear in most others. These details have been added and the incubation times clarified in the methods section.

Lines 241-242: in infected cells? Should mention this clearly.

Thank you. This point has been clarified in the text.

The effect of the Axl inhibitor on infection of HCC1819 (Fig.5D) is puzzling given that these cells do not seem to express Axl (Fig.S5). It is even more surprising that these cells support infection at all since Ace2 is barely expressed as well. Is it only a lack of surface expression or a lack of expression overall? The authors should discuss this discrepancy.
Also, the legend states H1819 instead of HCC1819.

We acknowledge that the H1819 findings are confusing and would require further experimentation. Thus, we agree that the data are a distraction. As a consequence, we have removed this dataset from the manuscript.
